# New Evidence of Skin Color Bias and Health Outcomes Using Sibling Difference Models: A Research Note

**DOI:** 10.1007/s13524-018-0756-6

**Published:** 2019-01-09

**Authors:** Thomas Laidley, Benjamin Domingue, Piyapat Sinsub, Kathleen Mullan Harris, Dalton Conley

**Affiliations:** 1 Department of Sociology, New York University, New York, NY 10012 USA; 2 Graduate School of Education, Stanford University, Stanford, CA 94305 USA; 3 Department of Sociology, Princeton University, Princeton, NJ 08544 USA; 4 Department of Sociology, University of North Carolina at Chapel Hill, Chapel Hill, NC 27516 USA

**Keywords:** Colorism, Discrimination, Skin color, Stratification, Public health

## Abstract

In this research note, we use data from the National Longitudinal Study of Adolescent to Adult Health (Add Health) to determine whether darker skin tone predicts hypertension among siblings using a family fixed-effects analytic strategy. We find that even after we account for common family background and home environment, body mass index, age, sex, and outdoor activity, darker skin color significantly predicts hypertension incidence among siblings. In a supplementary analysis using newly released genetic data from Add Health, we find no evidence that our results are biased by genetic pleiotropy, whereby differences in alleles among siblings relate to coloration and directly to cardiovascular health simultaneously. These results add to the extant evidence on color biases that are distinct from those based on race alone and that will likely only heighten in importance in an increasingly multiracial environment as categorization becomes more complex.

**Electronic supplementary material:**

The online version of this article (10.1007/s13524-018-0756-6) contains supplementary material, which is available to authorized users.

## Introduction

Associations between darker skin tone and lower educational attainment, occupational status, residential stability, income, and wealth have long been observed even after factors such as parental socioeconomic status and ethnoracial affiliation are controlled for (Dixon and Telles 2017; Keith and Herring 1991; Monk 2014; Painter et al. 2016; South et al. 2005). Much of the work on the influence of skin color on social inequality has focused on Latin America because it has historically exhibited greater fluidity between racial groups compared with places such as the United States. For instance, Telles and Paschel (2014) used a unique data set that included self-reported racial affiliation as well as interviewers’ ratings of respondent complexion to examine how skin tone predicts self-identification in Brazil, Colombia, Panama, and the Dominican Republic. They found that complexion is largely orthogonal to racial identification among Dominicans but highly predictive among Panamanians, with Brazil and Colombia exhibiting intermediate color-race elasticity. The authors argued that each country’s unique historical and cultural context explains this divergence.

Within the literature on skin color in Latin America, Brazil is often of particular interest to researchers because it has long allowed for an intermediate “mixed” designation between black and white in government surveys, with this gradation acting as a proxy for complexion in most work. Telles and Lim (1998) used nationally representative Brazilian survey data and found a socioeconomic hierarchy based on racial classification in the expected direction, with whites earning more than browns, who in turn earn more than blacks. The authors also found that income inequality among subgroups is higher when using interviewer-reported race as opposed to self-reported racial affiliation, which suggests the importance of socially ascribed categorization. In their work on assortative mating in Brazil, Gullickson and Torche (2014) found a negative association between higher educational attainment and marrying spouses with darker skin color, suggesting a market exchange predicated on complexion. Using an analytic approach similar to our study, Marteleto and Dondero (2016) used Brazilian birth register data and found that differential skin color designations predict educational disparities, even among twins. Similarly, Schwartzman (2007) found that highly educated nonwhite parents in Brazil are more likely than their less-educated counterparts to categorize their children as white. These findings suggest that skin color, racial identification, and social status are often interconnected in Latin America.

Research in the United States has documented similar phenomena, whereby skin color and social position are closely linked. Using U.S. Census data from the late nineteenth and early twentieth centuries—which allowed for a “mulatto” designation—Saperstein and Gullickson (2013) found that occupational gains over time resulted in a greater likelihood of transitioning from the black to the mulatto category as judged by census-takers. In their work on recent immigrants, Painter et al. (2016) found that darker-skinned immigrants are less wealthy than their lighter-complected counterparts and that this association is independent of racial affiliation (i.e., it operates both *within* and *across* discrete race categories). Vargas et al. (2016), however, found that skin tone is not associated with perceived discrimination among Latinx populations in the United States (both native and immigrant); importantly, though, skin complexion is self-rated in their data set.

Since the seminal work of Harburg et al. (1978), research has examined the links between complexion and biophysical outcomes, such as blood pressure. Analysts have found associations between darker skin tone and higher blood pressure even among high-income blacks, which is consistent with research finding weaker health returns to higher socioeconomic status among blacks compared with whites (Boen 2016; Colen et al. 2018; Riddell et al. 2017; but see also Do et al. 2012), along with evidence that perceived discrimination positively predicts hypertension, further suggesting its potential salience as a mechanism (Sims et al. 2012). Recent work similarly has found associations among darker skin tone, higher levels of perceived discrimination, and higher levels of depression and worse self-rated mental health among blacks. Moreover, intraracial health differences predicted by gradations in complexion were often at least as large as those found between blacks and whites more generally (Monk 2015). Other work has found that multiracial respondents who select white as the category that best describes them in addition to other backgrounds have reported worse self-rated health profiles than those who self-describe as white only. This result, however, is largely driven by the tendency for Native Americans—who have measurably worse health profiles—to best describe themselves as white but still multiracial (Bratter and Gorman 2011).

Although on balance the body of literature on skin tone has offered suggestive evidence that color-based discrimination (*colorism*) directly affects outcomes independent of racial or ethnic affiliation, the cross-sectional nature of most extant work and the possibility of endogeneity largely precludes drawing causal inferences. For example, genetic evidence confirms that African Americans who remained in the South after the Great Migration had less European ancestry—and, presumably, darker skin—than their peers who moved north (Baharian et al. 2016). If residence in the South causes hypertension through diet or another behavioral channel that cannot be realistically captured by customary data sources, darker skin tone—as a rough proxy for region—may predict worse health outcomes when it is merely associated with the true cause(s). Region is, of course, but one potential confounder. Although other work has used within-family fixed-effects (FE) methodology (Marteleto and Dondero 2016), included indicators of complexion rather than discrete racial classification (Monk 2014), and sought to examine health outcomes patterned on these distinctions (Monk 2015), we make a novel contribution to the literature on colorism by combining these features. Moreover, we present supplementary evidence that the genetic architecture of skin tone does not likely act as a source of bias by also directly affecting the health outcome.

## Methods

We use a restricted access file within the National Longitudinal Study of Adolescent to Adult Health (Add Health) that identifies genetic links between individual respondents. Add Health is a nationally representative, longitudinal study of adolescents in grades 7–12 in the fall of 1994 to the spring of 1995 academic year. To minimize the influence of contextual factors that may be implicated in discordant outcomes in well-being—that is, household, neighborhood, and school environments—we restrict our analyses to full siblings and use a family FE analytic approach to examine associations between our predictors and outcomes of interest within family units. (We produce estimates based on an expanded sample that includes twins in addition to full siblings in the online appendix, Table A1.) We use measures of interviewer-reported skin tone (an ordinal scale of 1–5)—measured only in Wave III (2001–2002), when respondents were about 18–26 years of age—to predict ever having been diagnosed with hypertension in Wave IV (self-reported in 2008), when respondents were about 24–32 years old, with other indicators constructed from supplementary biometric data.

The constructed hypertension indicators are derived from a combination of self-reports and blood pressure measurements collected in Wave IV, whereby readings over relevant thresholds would result in respondents being coded as positive for hypertension, even if they had indicated no formal medical diagnosis. Our main results are based on the total sample of full siblings from any background as well as a black/Latinx subgroup, which we might expect to exhibit more pronounced associations between complexion and health outcomes (although research is lacking on whether this may operate similarly in other racial or ethnic categories). In Add Health, respondents are free to check any racial affiliation that applies to them rather than a *best overall* category. As a result, by using a black/Latinx subsample, we include multiracial respondents in this grouping. We present results stratified on more rigid ethnoracial lines (i.e., white only, black only, and so on) in the online appendix (Table A4).

We find within-family discordance in skin tone and self-reported hypertension to be rather common in our data; about 21 % of the overall sample and 38 % of black/Latinx subsample siblings were recorded as having different skin tones (intraclass correlation coefficients (ICC) = .860 and .764, respectively). For hypertension self-reports, about 19 % of sibling groups exhibited Wave IV discordance in high blood pressure diagnosis both in the full and black/Latinx subsamples. We control for sex (because some research has indicated that among certain populations, females tend to have lighter skin than men; Jablonski and Chaplin 2000), age, and body mass index (BMI) collected in Wave IV. We also control for self-reported time outdoors in the summer months in Wave IV given that this factor possibly relates both to variations in complexion and to behaviors that may be systematically related to health outcomes and thus may be a confounding influence. Because skin tone was assessed years before hypertension, interviewers could not be primed to report darker skin based on blood pressure readings or medical diagnoses. We detail the variables in the analyses in Table 1; we include standard ordinary least squares (OLS) and logistic regression estimates in the online appendix (Table A6).

**Table 1:** Variables and descriptive statistics (full sibling sample)

	Question Text	Variable	Wave	Mean	SD	*N*
Hypertension	Has a doctor, nurse or other health care provider ever told you that you have or had: high blood pressure or hypertension? [If female add, when you were not pregnant] 0 = No, 1 = Yes	H4ID5C	IV	.105	.307	1,879
Hypertension, Stage 1	Self-reported hypertension, with measured blood pressure over Stage 1 thresholds in the biometric component replacing otherwise negative indicators; 0 = No, 1 = Yes	C4VAR045	IV	.236	.424	1,879
Hypertension, Stage 2	Self-reported hypertension, with measured blood pressure over Stage 2 thresholds in the biometric component replacing otherwise negative indicators; 0 = No, 1 = Yes	C4VAR046	IV	.123	.329	1,879
Skin Tone	What is the respondent’s skin color? [Interviewer coded] 1–5 (Recoded from original variable as: 1 = white; 2 = light brown; 3 = medium brown; 4 = dark brown; 5 = black)	H3IR17	III	4.350	1.126	1,630
Sex	Respondent’s biological sex [Interviewer coded and asked if necessary] 0 = Male, 1 = Female	BIO_SEX4	IV	.518	.500	1,879
Age	Age derived from date of birth at administration of Wave IV interview	H4OD1Y	IV	28.946	1.748	1,879
Time Outdoors	During a typical summer week, how many hours do you spend outdoors in the sun during the day?	H4DA17	IV	14.936	17.211	1,830
Body Mass Index	Body mass index calculated from height and weight in the biometric component of the survey	H4BMI	IV	29.175	7.742	1,847

## Results

Naive OLS and logistic regression estimates (online appendix, Table A6) show associations between darker skin tone and self-reported hypertension diagnosis that are significant at the *p* < .10 level among the black/Latinx subsample (*N* = 446). We find similar results (*p* < .05; *N* = 446) using Stage 2 hypertension as outcome, constructed from both self-reports and biometric measures taken by Add Health at Wave IV, but not for the Stage 1 indicator. Our preferred linear FE estimates (Table 2)—which account for family background and household environment, BMI, age, sex, and time outdoors—show that darker skin color positively and significantly predicts every hypertension outcome among our black/Latinx subsample (*N* = 446). (We stratify results based on discrete racial categories in the online appendix, Table A4.) Our FE conditional logit results based on the same subsample, which model hypertension as a binary outcome, offer similar estimates, although the Stage 2 outcome is significant only at the *p* < .10 level (*p* = .059). Estimates from our preferred linear specifications for self-reported hypertension suggest that each unit darker complexion—for example, going from *medium brown* to *dark brown*—is associated with a nearly 9 % increase in the probability of ever having been diagnosed, with similar effect sizes for constructed Stage 1/2 outcomes. In addition to our main analyses that use an ordinal scale of tone as outcome, we also construct a binary measure of skin complexion based on this scale (1 = black or dark brown; 0 = white, light brown, or medium brown) and find similar results, except for the linear FE specification modeling constructed Stage 1 hypertension as the outcome (online appendix, Table A2).

**Table 2:** Main results: Hypertension predicted by skin tone among full siblings

	Hypertension Self-report				Hypertension Constructed (Stage 1)				Hypertension Constructed (Stage 2)			
	Fixed Effects		Fixed Effects, Conditional Logit		Fixed Effects		Fixed Effects, Conditional Logit		Fixed Effects		Fixed Effects, Conditional Logit	
Darker Tone	0.043	0.088*	0.388	0.867*	0.063^†^	0.091*	0.413^†^	0.824*	0.051	0.094*	0.410	0.706^†^
	(0.030)	(0.038)	(0.315)	(0.439)	(0.035)	(0.042)	(0.247)	(0.380)	(0.031)	(0.039)	(0.294)	(0.374)
Age	0.010	0.020	0.153^†^	0.158	0.016^†^	0.044**	0.118*	0.383**	0.012^†^	0.025	0.164*	0.218^†^
	(0.006)	(0.013)	(0.081)	(0.146)	(0.009)	(0.017)	(0.058)	(0.121)	(0.007)	(0.014)	(0.074)	(0.127)
Female	–0.031	0.012	–0.419	0.234	–0.135***	–0.138^†^	–0.921***	–1.329**	–0.041	0.003	–0.551^†^	–0.209
	(0.026)	(0.052)	(0.321)	(0.658)	(0.036)	(0.071)	(0.238)	(0.505)	(0.028)	(0.058)	(0.304)	(0.547)
Time Outdoors	<–0.001	–0.001	–0.006	–0.008	<–0.001	0.001	–0.003	0.005	<0.001	<–0.001	–0.003	<0.001
	(0.001)	(0.002)	(0.009)	(0.016)	(0.001)	(0.003)	(0.007)	(0.015)	(0.001)	(0.003)	(0.008)	(0.015)
BMI	0.009***	0.011*	0.094***	0.067*	0.012***	0.016**	0.069***	0.102***	0.010***	0.011*	0.094***	0.066*
	(0.002)	(0.005)	(0.023)	(0.032)	(0.003)	(0.005)	(0.016)	(0.031)	(0.002)	(0.005)	(0.022)	(0.030)
Full Sample	✓		✓		✓		✓		✓		✓	
Black/Latinx		✓		✓		✓		✓		✓		✓
*F*	5.12***	2.35*	––	––	9.14***	6.24***	––	––	6.01***	2.52*	34.64***	13.01*
Chi-Square	––	––	29.46***	12.71*	––	––	48.53***	29.93***	––	––	––	––
*N*	1,496	446	268	102	1,496	446	477	160	1,496	446	306	119

*Notes:* Robust standard errors are shown in parentheses (OLS/FE specifications). For logit and conditional logit specifications, we present odds ratios and robust standard errors.

^†^*p* < .10; *p < .05; **p < .01; ***p < .001

The sibling FE analytic approach ostensibly accounts for a common family environment, but our inability to restrict our sample to monozygotic twins precludes us from wholly holding genetic background constant—although of course, full siblings do share a substantial amount of their genetic architecture. (We use an expanded sample that includes twins in Table A1 of the online appendix and find substantively similar results.) Genes that predict both complexion and hypertension risk may vary between full siblings and dizygotic twins (i.e., genetic pleiotropy). In this case, our estimates would potentially be biased by not accounting for a possible biologically endogenous genetic link between complexion and hypertension. We perform a robustness check using newly available supplementary genetic data in Add Health (McQueen et al. 2015) to determine whether several single nucleotide polymorphisms (SNPs) related to skin and eye coloration predict skin tone and hypertension (see Table A7 and Table A8 in the online appendix for, respectively, details on the SNPs and full results).

We perform this analysis among non-Hispanic whites, where such alleles are intuitively less likely to cause colorist discrimination—although research on the effect of complexion among whites is lacking—and thus any residual association with hypertension likely works directly through endogenous biochemical pathways. Neither PC1 (an indicator of African ancestry and darker skin) nor five of the six skin tone–related SNPs predict hypertension. Only the rs12913832 SNP significantly predicts hypertension; however, this is primarily an eye color allele (secondarily associated with complexion in prior work), does not predict skin tone in our data, and does not display variation in nonwhite populations, making it an unlikely candidate to explain sibling differences in hypertension within black/Latinx subgroups. Although there may be other pleiotropic alleles that we have not tested, and although the linkage patterns between the SNPs and cardiovascular-related causal alleles may differ in the black and non-Hispanic white populations, we nonetheless believe the weight of evidence suggests that the effects shown in the main analysis are more likely the result of social pathways than within-family variation in genetic background.

## Discussion

In our main results, skin tone significantly predicts hypertension outcomes only in the black/Latinx subsample. Our use of interviewer-coded skin color (to which we are limited given data availability) is thus a consideration when interpreting our results. One possibility is that skin tone is rated differently in a systematic way that could bias results—say, if white interviewers were more likely to interview siblings in more affluent settings, which could relate to their health profiles, while simultaneously coding them as lighter or darker than a black interviewer would. Indeed, Hill (2002) found that different-raced interviewers coded respondents’ complexion in systematically different ways, with white interviewers tending to rate blacks as darker than their counterparts. A related concern is the possibility that blood pressure itself is affected by interviewer race discordance, or a variation on the *white coat* effect in which the very act of clinical measurement induces temporary increases. When we stratify by interviewer race discordance (i.e., two siblings being interviewed by white and black field workers), however, we find that our results are concentrated in same-race interviewer dyads or triads, and thus this is not a compelling potential source of bias (online appendix, Table A5). More straightforwardly objective measures of complexion (i.e., using spectrophotometric instruments) or self-reported coloration (possibly reflecting internalized ideas of social status) may also offer different results. In one of the only studies we are aware of that employed both interviewer-coded and self-reported skin tone as predictors, Monk (2015) found that self-reported complexion measures significantly predicted self-reported hypertension, while interviewer-coded measures failed to do so. Thus, it is possible that our estimates are actually conservative and that using self-reported skin tone would evince more robust associations. We also stratify on same- and discordant-sex sibling groupings (online appendix, Table A3) and find that our results are driven by opposite-sex siblings. We believe this is attributable the greater variation in tone within these families; we find ICCs of .68 and .81 in mixed- and opposite-sex sibships, respectively.

We also find that an ordinal scale commonly used to proxy discrimination in Add Health (0 = never, 3 = often)—“How often do you feel you have been treated with less respect or courtesy than other people?”—did not predict hypertension in our models when added, and did not appreciably alter the results for skin tone. A follow-up question specifically asked participants what this disrespect could be attributable to, and merely 1.6 % reported skin color, with an additional 8.9 % citing race. When we construct a dichotomous indicator of whether respondents indicated past discrimination based on either race or color (we cannot use the latter on its own because of the rarity of the response) and include it in our models, the results are substantively similar for tone, while the discrimination indicator is significant at *p* < .10 but predicts less hypertension, not more. We believe that the direction of the coefficient is likely an artifact of how few observations indicate positive responses; only 31 of 546 total black/Latinx respondents said they were disrespected because of either skin color or racial background. Because of the relatively crude nature of the measure and what it conceptually conveys (i.e., *respect* and *courtesy*, with *discrimination* offered as a specific selection only if the respondent indicated *sometimes* or *often* on the first question, which likely explains why so few did), we argue that it does not discount the possibility that our results are operating through a social bias channel.

With respect to the links between discrimination and cardiovascular outcomes such as hypertension, recent research suggests that racial bias is associated with leukocyte telomere length (an indicator of general systemic aging; Chae et al. 2014) and C-reactive protein levels related to inflammation and cardiovascular health (Goosby et al. 2015). The results of these studies suggest that discrimination manifests in physiological responses that in turn affect cardiovascular outcomes, and may offer insights for the results we obtain here. With the increasing availability of supplementary biometric data, future research could model these processes in sketching out the specific mechanisms embedded in the relationship between coloration and well-being.

## Conclusion

In this article, we find that even among full siblings and after a range of relevant factors are controlled for, darker skin tone is associated with a greater likelihood of having been diagnosed with hypertension. This finding supports extant evidence of color bias, although with our data, we cannot determine whether this may be a function of inter- and/or intraracial processes, nor can we definitively rule out a more complex causal pathway that does not directly involve discrimination. We also offer additional evidence that these results are not likely to be entirely an artifact of genetic pleiotropy between skin tone and hypertension-causing alleles. To our knowledge, our study is the first to bring a sibling FE analytic strategy (as well as genetic data) to bear on examining health outcomes patterned on complexion to support a social explanation for why darker-skinned people disproportionately suffer from stress-related cardiovascular health issues. These findings provide yet more evidence that skin color should be considered as a relevant factor—independent of racial or ethnic affiliation alone, and particularly in an increasingly multiracial society—in reproducing pernicious inequalities in health and well-being.

## Electronic supplementary material

ESM 1 (PDF 241 kb)
