# Supplementary material for: New Evidence of Skin Color Bias and Health Outcomes Using Sibling Difference Models: A Research Note
Source: Demography. 2019 Jan 9;56(2):753–62. doi: 10.1007/s13524-018-0756-6 (PMC6449491; doi:10.1007/s13524-018-0756-6)
Supplement: Supplementary file 1 — (PDF 241 kb) [file 13524_2018_756_MOESM1_ESM.pdf]

Online Appendix

New Evidence of Skin Color Bias and Health Outcomes Using Sibling Difference Models: A Research Note

Contents

1. Table A1— Hypertension Predicted by Skin Tone among Both Full Siblings and Twins
2. Table A2— Main Results—Hypertension Predicted by Dichotomous Skin Tone Measure (0= Lighter Tone, 1=Darker) Among Both Full Siblings and Twins
3. Table A3— Main Results—Hypertension Predicted by Skin Tone among Same- and Opposite-Sex Sibship Groups (Large Sample)
4. Table A4— Main Results—Hypertension Predicted by Skin Tone among White and Black Only, Latinx Subsamples (Large Sample)
5. Table A5— Main Results Stratified by Interviewer Race Discordance
6. Table A6— OLS and Logit Results—Hypertension Predicted by Skin Tone
7. Table A7—Description of Skin and Eye Color SNPs
8. Table A8—Skin Tone and Hypertension Predicted by Alleles for Complexion and Eye Color

TABLE A1. Main Results—Hypertension Predicted by Skin Tone among Both Full Siblings and Twins

	Hypertension Self Report				Hypertension Constructed (Stage 1)				Hypertension Constructed (Stage 2)			
	FE		FE—Cond. Logit		FE		FE—Cond. Logit		FE		FE—Cond. Logit	
Darker Tone	.033	.070*	.271	.568*	.051	.072*	.348†	.545*	.033	.064†	.238	.466†
	(.025)	(.031)	(.232)	(.281)	(.030)	(.036)	(.194)	(.243)	(.026)	(.032)	(.201)	(.255)
Age	.012†	.020	.166*	.153	.016†	.039*	.119*	.268**	.013†	.020	.168*	.128
	(.006)	(.012)	(.079)	(.132)	(.009)	(.017)	(.057)	(.099)	(.007)	(.014)	(.068)	(.109)
Female	-.011	.070	-.150	.623	-.137***	-.084	-.917***	-.697*	-.022	.065	-.272	.390
	(.023)	(.044)	(.260)	(.444)	(.031)	(.059)	(.205)	(.346)	(.025)	(.050)	(.247)	(.381)
Outdoors	<-.001	<.001	.003	.005	<-.001	.001	-.004	.004	<.001	<.001	.002	<.001
	(<.001)	(.002)	(.007)	(.012)	(.001)	(.002)	(.006)	(.010)	(.001)	(.002)	(.007)	(.012)
BMI	.007***	.007†	.078***	.043†	.012***	.012**	.077***	.082**	.009***	.007	.082***	.039†
	(.002)	(.003)	(.019)	(.026)	(.002)	(.004)	(.014)	(.025)	(.002)	(.004)	(.020)	(.024)
Full Sample	✓		✓		✓		✓		✓		✓	
Black/Latinx		✓		✓		✓		✓		✓		✓
F	4.78***	2.35*	—	—	12.39***	5.21***	—	—	5.73***	1.84	—	—
X^2	—	—	27.78***	13.09*	—	—	65.27***	25.52***	—	—	23.03***	10.18†
N	2333	780	392	160	2333	780	695	253	2333	780	448	183

NOTE: Robust standard errors in parentheses (OLS/FE specifications). For logit and conditional logit specifications, we give odds ratios and robust standard errors.

† $p < .1$, * $p < .05$, ** $p < .01$, *** $p < .001$

TABLE A2. Main Results—Hypertension Predicted by Dichotomous Skin Tone Measure (0= Lighter Tone, 1=Darker) Among Both Full Siblings and Twins

	Hypertension Self Report				Hypertension Constructed (Stage 1)				Hypertension Constructed (Stage 2)			
	FE		FE—Cond. Logit		FE		FE—Cond. Logit		FE		FE—Cond. Logit	
Darker Tone	.101†	.134*	.844	1.349*	.066	.093	.545	.968†	.097	.120†	.760	.976†
	(.059)	(.062)	(.573)	(.652)	(.064)	(.067)	(.480)	(.563)	(.064)	(.069)	(.502)	(.543)
Age	.012†	.018	.176*	.204	.016†	.037*	.119*	.266**	.013†	.019	.179*	.163
	(.006)	(.012)	(.080)	(.138)	(.009)	(.017)	(.057)	(.099)	(.007)	(.014)	(.073)	(.113)
Female	-.012	.065	-.158	.596	-.142***	-.093	-.951***	-.753*	-.023	.060	-.282	.359
	(.023)	(.044)	(.259)	(.440)	(.031)	(.059)	(.204)	(.343)	(.025)	(.050)	(.243)	(.379)
Outdoors	<-.001	<.001	.003	.007	<-.001	.001	-.004	.003	<.001	<.001	.002	.003
	(.001)	(.002)	(.007)	(.012)	(.001)	(.002)	(.006)	(.010)	(.001)	(.002)	(.007)	(.013)
BMI	.007***	.007†	.075***	.036	.012***	.012**	.075***	.075**	.008***	.006	.080***	.035
	(.002)	(.004)	(.019)	(.026)	(.002)	(.004)	(.014)	(.024)	(.002)	(.004)	(.018)	(.024)
Full Sample	✓		✓		✓		✓		✓		✓	
Black/Latinx		✓		✓		✓		✓		✓		✓
F	4.91***	2.20†	—	—	11.99***	4.55***	—	—	5.80***	1.67	—	—
X^2	—	—	28.71***	13.35*	—	—	63.22***	23.04***	—	—	34.55***	10.01†
N	2333	780	392	160	2333	780	695	253	2333	780	448	183

NOTE: Robust standard errors in parentheses (OLS/FE specifications). For logit and conditional logit specifications, we give odds ratios and robust standard errors. The dichotomous skin tone measure (lighter tone) is coded 0 if the respondent is rated 1 or 2 in complexion (Black or dark brown), 1 if 3 through 5 (medium brown, light brown, and white).

† $p < .1$, * $p < .05$, ** $p < .01$, *** $p < .001$

TABLE A3. Main Results—Hypertension Predicted by Skin Tone among Same- and Opposite-Sex Sibship Groups (Large Sample)

	Self-Reported Hypertension Diagnosis				Hypertension, Constructed (Stage 1)				Hypertension, Constructed (Stage 2)			
	FE—Same		FE—Opposite		FE—Same		FE—Opposite		FE—Same		FE—Opposite	
Darker Tone	.012	.037	.057	.113*	.037	.031	.068	.123*	.016	.039	.053	.095*
	(.032)	(.043)	(.040)	(.045)	(.038)	(.046)	(.046)	(.052)	(.033)	(.044)	(.042)	(.047)
Age	.013	.032*	.011	.006	.019	.046*	.016	.027	.012	.019	.016	.017
	(.008)	(.016)	(.009)	(.018)	(.012)	(.022)	(.014)	(.024)	(.010)	(.019)	(.010)	(.020)
Female	—	—	-.004	.085†	—	—	-.117***	-.048	—	—	-.010	.088
			(.024)	(.044)			(.032)	(.060)			(.026)	(.050)
Outdoors	<-.001	.001	.001	<.001	-.002†	-.001	.002†	.003	-.001	-.001	.002	.001
	(.001)	(.002)	(.001)	(.002)	(.001)	(.002)	(.001)	(.003)	(.001)	(.002)	(.001)	(.003)
BMI	.008**	.012†	.006*	.003	.016***	.019**	.009**	.006	.010***	.013†	.007**	.001
	(.003)	(.006)	(.002)	(.005)	(.003)	(.006)	(.003)	(.006)	(.003)	(.007)	(.003)	(.005)
Full Sample	✓		✓		✓		✓		✓		✓	
Black/Latino		✓		✓		✓		✓		✓		✓
F	3.47**	2.47*	2.40*	1.94†	7.92***	5.10***	7.40***	2.56*	4.15**	1.56	2.90*	1.33
N	1415	468	918	312	1415	468	918	312	1415	468	918	312

NOTE: Robust standard errors in parentheses (OLS/FE specifications). For logit and conditional logit specifications, we give odds ratios and robust standard errors.

† $p < .1$, * $p < .05$, ** $p < .01$, *** $p < .001$

TABLE A4. Main Results—Hypertension Predicted by Skin Tone among White and Black Only, Latinx Subsamples (Large Sample)

	Self-Reported (Linear FE)			Constructed, Stage I (Linear FE)			Constructed, Stage II (Linear FE)		
Darker Tone	.014	.083†	.080	.116†	.069	.136*	.050	.059	.079
	(.049)	(.044)	(.056)	(.062)	(.052)	(.062)	(.074)	(.045)	(.056)
Age	.012	.030	.012	.012	.070**	.008	.015	.031	.005
	(.008)	(.022)	(.006)	(.012)	(.025)	(.023)	(.009)	(.024)	(.015)
Female	-.041	.101	.085	-.182***	.012	-.137	-.073*	.115	.046
	(.030)	(.067)	(.065)	(.043)	(.086)	(.096)	(.031)	(.078)	(.075
Outdoors	<-.001	.001	-.001	<-.002	.002	-.002	<.002	.001	-.001
	(.001)	(.003)	(.003)	(.001)	(.003)	(.003)	(.001)	(.003)	(.003)
BMI	.007**	.007	.010†	-.182***	.007	.023**	.009***	.004	.012†
	(.002)	(.006)	(.006)	(.043)	(.006)	(.007)	(.002)	(.006)	(.012)
White only	✓			✓			✓		
Black only		✓			✓			✓	
Latinx, any race			✓			✓			✓
F	3.25**	1.55	1.39	8.17***	2.40*	4.15**	4.76***	1.09	1.15
N	1340	431	335	1340	416	335	1340	416	335

NOTE: Robust standard errors in parentheses (OLS/FE specifications). For logit and conditional logit specifications, we give odds ratios and robust standard errors.
† $p < .1$, * $p < .05$, ** $p < .01$, *** $p < .001$

TABLE A5. Main Results Stratified by Interviewer Race Discordance

	Self-Reported Hypertension				Constructed (Hypertension Stage I)				Contstructed (Hypertension Stage II)			
Darker Tone	.062*	-.071	.092*	-.068	.076*	.032	.104*	.006	.042	-.033	.062	-.007
	(.028)	(.058)	(.037)	(.078)	(.037)	(.076)	(.049)	(.080)	(.029)	(.067)	(.038)	(.098)
Age	.014†	.007	.015	-.039	.017	-.013	.035†	-.063	.014†	-.003	.014	-.007
	(.007)	(.017)	(.016)	(.025)	(.010)	(.026)	(.020)	(.041)	(.008)	(.024)	(.017)	(.012)
Female	-.015	.049	.078	.111	-.136***	-.134†	-.065	-.092	-.028	.027	.075	.103
	(.026)	(.054)	(.058)	(.083)	(.036)	(.079)	(.075)	(.125)	(.028)	(.059)	(.065)	(.078)
Outdoors	.001	.003†	.002	.001	<-.001	.006**	.001	.010	.001	.003	.001	<.001
	(.001)	(.002)	(.002)	(.002)	(.001)	(.002)	(.002)	(.003)	(.006)	(.002)	(.002)	(.002)
BMI	.006**	.009	.007	.002	.012***	.015*	.012*	.013	.008***	.010	.005	.007
	(.002)	(.006)	(.004)	(.010)	(.003)	(.006)	(.005)	(.008)	(.002)	(.007)	(.005)	(.098)
Full Sample	✓	✓			✓	✓			✓	✓		
Black/Latinx			✓	✓			✓	✓			✓	✓
Interviewers Same Race	Yes	No	Yes	No	Yes	No	Yes	No	Yes	No	Yes	No
F	4.50***	1.44	2.21†	.96	9.29***	3.55**	3.53**	2.94*	4.97***	.86	1.09	1.00
N	1600	248	489	99	1600	248	489	99	1600	248	489	99

NOTE: Robust standard errors in parentheses (OLS/FE specifications).
† $p < .1$, * $p < .05$, ** $p < .01$, *** $p < .001$

TABLE A6. OLS and Logit Results—Hypertension Predicted by Skin Tone

	Hypertension Self Report				Hypertension Constructed (Stage 1)				Hypertension Constructed (Stage 2)			
	OLS		Logit		OLS		Logit		OLS		Logit	
Darker Tone	.004	.015†	.057	.175†	.010	.018	.066	.104	.011	.023*	.106†	.219*
	(.007)	(.009)	(.067)	(.093)	(.009)	(.014)	(.053)	(.079)	(.007)	(.010)	(.062)	(.090)
Age	.012**	.013†	.139**	.154†	.018**	.029**	.108**	.168**	.012	.014†	.126**	.143†
	(.004)	(.008)	(.048)	(.093)	(.006)	(.011)	(.036)	(.063)	(.004)	(.008)	(.045)	(.081)
Female	-.042**	-.062*	-.491**	-.758*	-.161***	-.174***	-.993***	-1.013***	-.059***	-.072*	-.607***	-.757**
	(.015)	(.028)	(.170)	(.310)	(.021)	(.039)	(.128)	(.226)	(.016)	(.030)	(.159)	(.284)
Outdoors	<-.001	-.001	-.005	-.015	<-.001	.001	-.001	.003	<-.001	<-.001	-.004	-.011
	(<.001)	(.001)	(.005)	(.012)	(.001)	(.001)	(.003)	(.007)	(<.001)	(.001)	(.004)	(.010)
BMI	.009***	.011***	.075***	.105***	.014***	.015***	.077***	.084***	.010***	.012***	.075***	.104***
	(.001)	(.002)	(.010)	(.018)	(.001)	(.003)	(.009)	(.014)	(.001)	(.002)	(.010)	(.017)
Full Sample	✓		✓		✓		✓		✓		✓	
Black/Latino		✓		✓		✓		✓		✓		✓
F	13.48***	6.81***	—	—	37.96***	14.82***	—	—	16.35***	8.43***	—	—
X^2	—	—	70.98***	38.19***	—	—	139.15***	56.28***	—	—	78.28***	43.78***
N	1648	504	1648	504	1648	504	1648	504	1648	504	1648	504

NOTE: Robust standard errors in parentheses.
† $p < .1$, * $p < .05$, ** $p < .01$, *** $p < .001$

TABLE A7. Description of Skin and Eye Color SNPs

SNP	Description	Reference
rs16891982	Predicts skin and hair color, and melanoma susceptibility in European populations	Soejima & Koda (2007)
rs1545397	Predicts non-dark (i.e. light or medium) skin coloration	Hart et al. (2013)
rs12913832	Predicts eye coloration; variations relatively common in groups with European ancestry but rare in other racial groups	Elberg et al. (2008)
rs1426654	Influences skin coloration and can be used to categorize ancestry as either European, African, or Asian	Giardina et al. (2008)
rs885479	Influences skin coloration, i.e. between light and non-light (medium or dark)	Hart et al. (2013)
rs6119471	Influences skin coloration, i.e. between light and non-light (medium or dark)	Hart et al. (2013)

References

Elberg H, Troelsen J, Nielsen M, Mikkelsen A, Mengel-From J, Kiaer KW, Hansen L. Blue eye color in humans may be caused by a perfectly associated founder mutation in regulatory element located within the HERC2 gene inhibiting OCA2 expression. *Hum Genet*. 2008; 123: 177-187.

Giardina E, Pietrangeli I, Martínez-Labarga C, Martone C, de Angelis F, Spinella A, De Stefano G, Rickards O, Novelli G. Haplotypes in SLC24A5 gene as ancestry informative markers in different populations. *Curr Genomics*. 2008: 9: 110-114.

Hart KL, Kimura SL, Mushailov V, Budimlija ZM, Prinz M, Wurmbach E. Improved eye- and skin-color prediction based on 8 SNPs. *Croat Med J*. 2013; 54: 248-256.

Soejima M, Koda Y. Population differences of two coding SNPs in pigmentation-related genes *SLC24A5* and *SLC45A2*. *Int J Legal Med*. 2007; 121: 36-39.

TABLE A8. Skin Tone and Hypertension Predicted by Alleles for Complexion and Eye Color

	Outcome: Skin Tone						Outcome: Hypertension					
	(1)	(2)	(3)	(4)	(5)	(6)	(7)	(8)	(9)	(10)	(11)	(12)
PC1	-28.339		-38.097†	-28.140		-38.324†	23.752		41.050	28.862		54.339
	(18.761)		(21.645)	(19.393)		(22.528)	(29.777)		(33.917)	(31.593)		(36.550)
rs16891982		-.019	-.008		-.021	-.010		-.039	-.049		-.081	-.096
		(.043)	(.043)		(.043)	(.044)		(.065)	(.066)		(.070)	(.071)
rs154397		.001	.006		.005	.009		-.003	-.008		.007	.001
		(.033)	(.033)		(.033)	(.033)		(.051)	(.051)		(.054)	(.054)
rs12913832		-.014	-.012		-.014	-.012		.055*	.052*		.054*	.051
		(.016)	(.016)		(.016)	(.016)		(.026)	(.026)		(.027)	(.027)
rs1426654		.023	.033		.015	.026		-.132	-.144		-.132	-.149
		(.190)	(.190)		(.193)	(.193)		(.311)	(.311)		(.312)	(.312)
rs885479		-.003	-.006		-.003	-.006		-.006	-.002		-.009	-.005
		(.024)	(.024)		(.024)	(.024)		(.038)	(.038)		(.039)	(.039)
rs6119471		.032	.054		.016	.038		-.099	-.122		-.073	-.104
		(.136)	(.136)		(.138)	(.139)		(.223)	(.224)		(.224)	(.225)
Female				-.012	-.015	-.012	-.078***	-.076**	-.079**	-.080**	-.076**	-.080**
				(.016)	(.016)	(.016)	(.024)	(.024)	(.024)	(.025)	(.026)	(.026)
Birth Year				-.010*	-.010*	-.010*	-.006	-.007	-.007	-.004	-.005	-.005
				(.004)	(.005)	(.005)	(.007)	(.007)	(.007)	(.007)	(.007)	(.007)
Outside				<.001	<.001	<.001	-.001	<-.001	<-.001	<-.001	<-.001	<-.001
				(<.001)	(<.001)	(<.001)	(.001)	(<.001)	(<.001)	(<.001)	(<.001)	(<.001)
Darker Tone										.051	.051	.044
										(.064)	(.065)	(.065)
Intercept	4.782***	4.977***	4.720***	5.542***	5.733***	5.473***	.782	.666	.941	.903	.765	1.095
	(.126)	(.009)	(.147)	(.375)	(.359)	(.389)	(.576)	(.548)	(.593)	(.707)	(.688)	(.723)
N	664	658	658	643	658	637	720	713	713	643	637	637
r^2	.003	.002	.006	.012	.010	.015	.017	.024	.026	.017	.025	.029
